# 2,7-Dihydr­oxy-3,6-dimethoxy­phenanthrene from *Dehaasia longipedicellata*

**DOI:** 10.1107/S1600536808014451

**Published:** 2008-05-21

**Authors:** Mat Ropi Mukhtar, Mohd Azlan Nafiah, Khalijah Awang, A. Hamid A. Hadi, Seik Weng Ng

**Affiliations:** a Department of Chemistry, University of Malaya, 50603 Kuala Lumpur, Malaysia

## Abstract

The hydr­oxy groups in the title compound, C_16_H_14_O_4_, are each hydrogen bonded to the adjacent meth­oxy O atom; one of the two hydr­oxy groups is additionally linked to the O atom of the meth­oxy group of another mol­ecule, forming a linear chain.

## Related literature

For related compounds isolated from other plants, see: Bhandari *et al.* (1985 ▶); Mujumder *et al.* (1985 ▶); Theuns *et al.* (1985 ▶); Zurinah Mahmud *et al.* (1992 ▶). For the crystal structure of 2,3-dimeth­oxy-6,7-methyl­enedioxy­phenanthrene, see: Wang *et al.* (2007 ▶).
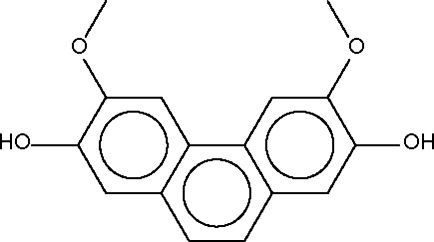


## Experimental

### 

#### Crystal data

C_16_H_14_O_4_
*M* *_r_* = 270.27
Monoclinic, 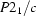

*a* = 11.6268 (2) Å
*b* = 7.2207 (1) Å
*c* = 16.5351 (2) Å
β = 109.196 (1)°
*V* = 1311.00 (3) Å^3^
*Z* = 4
Mo *K*α radiation
μ = 0.10 mm^−1^
*T* = 100 (2) K
0.30 × 0.25 × 0.05 mm

#### Data collection

Bruker SMART APEX diffractometer
Absorption correction: none
15710 measured reflections
2991 independent reflections
2671 reflections with *I* > 2σ(*I*)
*R*_int_ = 0.020

#### Refinement

*R*[*F* ^2^ > 2σ(*F* ^2^)] = 0.046
*wR*(*F* ^2^) = 0.150
*S* = 1.10
2991 reflections
237 parameters
14 restraints
All H-atom parameters refined
Δρ_max_ = 0.42 e Å^−3^
Δρ_min_ = −0.23 e Å^−3^

### 

Data collection: *APEX2* (Bruker, 2007 ▶); cell refinement: *SAINT* (Bruker, 2007 ▶); data reduction: *SAINT*; program(s) used to solve structure: *SHELXS97* (Sheldrick, 2008 ▶); program(s) used to refine structure: *SHELXL97* (Sheldrick, 2008 ▶); molecular graphics: *X-SEED* (Barbour, 2001 ▶); software used to prepare material for publication: *publCIF* (Westrip, 2008 ▶).

## Supplementary Material

Crystal structure: contains datablocks I, global. DOI: 10.1107/S1600536808014451/bt2712sup1.cif

Structure factors: contains datablocks I. DOI: 10.1107/S1600536808014451/bt2712Isup2.hkl

Additional supplementary materials:  crystallographic information; 3D view; checkCIF report

## Figures and Tables

**Table 1:** Hydrogen-bond geometry (Å, °)

*D*—H⋯*A*	*D*—H	H⋯*A*	*D*⋯*A*	*D*—H⋯*A*
O2—H2o⋯O1	0.85 (1)	2.20 (3)	2.670 (2)	115 (2)
O2—H2o⋯O3^i^	0.85 (1)	1.95 (1)	2.754 (2)	159 (3)
O3—H3o⋯O4	0.85 (1)	2.08 (3)	2.614 (2)	121 (3)

Symmetry code: (i) 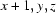.

**Fig. 1.**
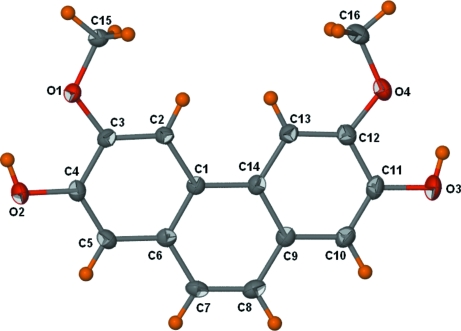
Displacement ellipsoid plot (Barbour, 2001) of the molecule of C16H16O4 drawn at the 70% probability level. Hydrogen atoms are shown as spheres of arbitrary radii.

**Fig. 2.**
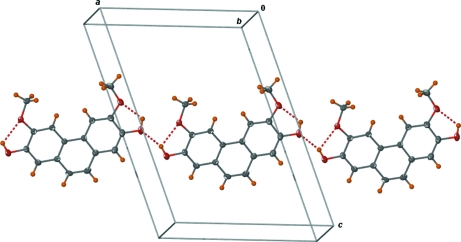
Hydrogen-bonded chain structure.

## supplementary crystallographic information

### Comment

2,7-Dihydroxy-3,6-dimethoxylphenanthrene (Scheme I, Fig. 1) is a new compound isolated from *Dehaasia longipedicellata* (Ridl.) Kosterm. The hydroxy groups   are each hydrogen-bonded to the adjacent methoxy oxygen; one of the two hydroxy groups is additionally linked to the oxygen atom of the methoxy group of another molecule to form a linear chain.

### Experimental

*Dehaasia longipedicellata* (Ridl.) Kosterm. was collected in Raub Forest Reserve, Pahang, Malaysia, in 1997. Specimens (KL4719) were deposited at the herbarium, Department of Chemistry, University of Malaya and the herbarium of the Forest Research Institute of Malaysia.

Some 1.4 kg of dried and ground leaves of *D. longipedicellata* was extracted with dichloromethane. The dichloromethane extract was concentrated under reduced pressure to a volume of 500 ml. This was repeatedly extracted with a solution of 5% hydrochloric acid. The combined extracts were then basified with 10% ammonium hydroxide to pH 11 and then re-extracted with dichloromethane. The brown alkaloid fraction amounted to (8.83 g). A portion (3 g) was subjected to column chromatography on silica gel 60 GF_254 _by using a step gradient of dichloromethane and methanol. The separation afforded 15 fractions, the first (100% dichloromethane) gave 2,7-dihydroxy-3,6-dimethoxyphenanthrene (8 mg), whose formulation was established by spectroscopic analysis. Light brown prisms were obtained upon recrystallization from dichloromethane.

### Refinement

Hydrogen atoms were located in a difference Fourier map. They were refined isotropically with distance restraints of C–H 0.95±0.01 Å and O–H 0.85±0.01 Å.

### Crystal data

**Table:**

C_16_H_14_O_4_	*F*_000_ = 568
*M**_r_* = 270.27	*D*_x_ = 1.369 Mg m^−^^3^
Monoclinic, *P*2_1_/*c*	Mo *K*α radiation λ = 0.71073 Å
Hall symbol: -P 2ybc	Cell parameters from 8700 reflections
*a* = 11.6268 (2) Å	θ = 2.6–28.3º
*b* = 7.2207 (1) Å	µ = 0.10 mm^−^^1^
*c* = 16.5351 (2) Å	*T* = 100 (2) K
β = 109.196 (1)º	Prism, pale brown
*V* = 1311.00 (3) Å^3^	0.30 × 0.25 × 0.05 mm
*Z* = 4	

### Data collection

**Table:**

Bruker SMART APEX diffractometer	2671 reflections with *I* > 2σ(*I*)
Radiation source: fine-focus sealed tube	*R*_int_ = 0.020
Monochromator: graphite	θ_max_ = 27.5º
*T* = 100(2) K	θ_min_ = 2.6º
ω scans	*h* = −11→15
Absorption correction: None	*k* = −9→9
15710 measured reflections	*l* = −21→21
2991 independent reflections	

### Refinement

**Table:**

Refinement on *F*^2^	Secondary atom site location: difference Fourier map
Least-squares matrix: full	Hydrogen site location: inferred from neighbouring sites
*R*[*F*^2^ > 2σ(*F*^2^)] = 0.046	All H-atom parameters refined
*wR*(*F*^2^) = 0.150	*w* = 1/[σ^2^(*F*_o_^2^) + (0.07*P*)^2^ + 1.2422*P*] where *P* = (*F*_o_^2^ + 2*F*_c_^2^)/3
*S* = 1.11	(Δ/σ)_max_ = 0.001
2991 reflections	Δρ_max_ = 0.42 e Å^−^^3^
237 parameters	Δρ_min_ = −0.23 e Å^−^^3^
14 restraints	Extinction correction: none
Primary atom site location: structure-invariant direct methods	

### Fractional atomic coordinates and isotropic or equivalent isotropic displacement parameters (Å^2^)

**Table:**

	*x*	*y*	*z*	*U*_iso_*/*U*_eq_	
O1	0.71900 (10)	0.23952 (17)	0.51347 (7)	0.0175 (3)	
O2	0.85160 (11)	0.40262 (19)	0.65922 (8)	0.0213 (3)	
O3	−0.02829 (12)	0.2974 (2)	0.54817 (9)	0.0277 (3)	
O4	0.05316 (11)	0.19182 (19)	0.42576 (8)	0.0236 (3)	
C1	0.47440 (14)	0.3578 (2)	0.60344 (10)	0.0137 (3)	
C2	0.53118 (14)	0.2890 (2)	0.54541 (10)	0.0141 (3)	
C3	0.65507 (14)	0.3029 (2)	0.56408 (10)	0.0147 (3)	
C4	0.72887 (14)	0.3887 (2)	0.64087 (10)	0.0160 (3)	
C5	0.67565 (15)	0.4568 (2)	0.69713 (10)	0.0164 (3)	
C6	0.54872 (14)	0.4405 (2)	0.68039 (10)	0.0148 (3)	
C7	0.49473 (15)	0.5060 (2)	0.74130 (10)	0.0161 (3)	
C8	0.37442 (15)	0.4861 (2)	0.72813 (10)	0.0174 (3)	
C9	0.29560 (15)	0.4047 (2)	0.65046 (10)	0.0159 (3)	
C10	0.16910 (16)	0.3874 (2)	0.63624 (11)	0.0197 (4)	
C11	0.09423 (15)	0.3145 (2)	0.56196 (11)	0.0197 (4)	
C12	0.14106 (15)	0.2572 (2)	0.49676 (11)	0.0180 (3)	
C13	0.26312 (15)	0.2727 (2)	0.50896 (10)	0.0157 (3)	
C14	0.34417 (14)	0.3444 (2)	0.58698 (10)	0.0142 (3)	
C15	0.64950 (15)	0.1659 (2)	0.43192 (10)	0.0172 (3)	
C16	0.09098 (17)	0.1296 (3)	0.35617 (11)	0.0234 (4)	
H2O	0.870 (2)	0.361 (4)	0.6173 (12)	0.042 (7)*	
H3O	−0.059 (3)	0.257 (4)	0.4974 (10)	0.060 (9)*	
H2	0.4840 (17)	0.236 (3)	0.4925 (9)	0.019 (5)*	
H5	0.7270 (16)	0.513 (3)	0.7485 (9)	0.019 (5)*	
H7	0.5458 (16)	0.571 (3)	0.7897 (10)	0.019 (5)*	
H8	0.3392 (18)	0.525 (3)	0.7698 (11)	0.019 (5)*	
H10	0.1355 (19)	0.429 (3)	0.6789 (11)	0.027 (6)*	
H13	0.2932 (18)	0.230 (3)	0.4656 (10)	0.019 (5)*	
H151	0.7096 (16)	0.132 (3)	0.4069 (14)	0.028 (6)*	
H152	0.5945 (16)	0.259 (2)	0.4015 (12)	0.019 (5)*	
H153	0.6033 (16)	0.058 (2)	0.4361 (13)	0.015 (5)*	
H161	0.0189 (14)	0.090 (3)	0.3123 (11)	0.030 (6)*	
H162	0.1507 (18)	0.034 (3)	0.3737 (15)	0.034 (6)*	
H163	0.128 (2)	0.234 (2)	0.3382 (15)	0.032 (6)*	

### Atomic displacement parameters (Å^2^)

**Table:**

	*U*^11^	*U*^22^	*U*^33^	*U*^12^	*U*^13^	*U*^23^
O1	0.0127 (5)	0.0242 (6)	0.0160 (6)	0.0004 (4)	0.0053 (4)	−0.0032 (4)
O2	0.0122 (6)	0.0314 (7)	0.0193 (6)	−0.0021 (5)	0.0038 (4)	−0.0035 (5)
O3	0.0126 (6)	0.0442 (8)	0.0273 (7)	−0.0017 (5)	0.0079 (5)	−0.0053 (6)
O4	0.0135 (6)	0.0342 (7)	0.0207 (6)	−0.0026 (5)	0.0025 (5)	−0.0061 (5)
C1	0.0140 (7)	0.0133 (7)	0.0138 (7)	0.0008 (6)	0.0046 (6)	0.0016 (5)
C2	0.0148 (7)	0.0151 (7)	0.0122 (7)	0.0001 (6)	0.0039 (6)	0.0004 (5)
C3	0.0152 (7)	0.0153 (7)	0.0143 (7)	0.0014 (6)	0.0058 (6)	0.0013 (6)
C4	0.0126 (7)	0.0174 (7)	0.0171 (7)	−0.0008 (6)	0.0037 (6)	0.0023 (6)
C5	0.0164 (8)	0.0169 (7)	0.0137 (7)	−0.0017 (6)	0.0019 (6)	−0.0004 (6)
C6	0.0163 (8)	0.0145 (7)	0.0132 (7)	0.0009 (6)	0.0043 (6)	0.0023 (5)
C7	0.0199 (8)	0.0154 (7)	0.0111 (7)	0.0007 (6)	0.0027 (6)	−0.0002 (5)
C8	0.0216 (8)	0.0177 (7)	0.0148 (7)	0.0033 (6)	0.0086 (6)	0.0013 (6)
C9	0.0157 (8)	0.0165 (7)	0.0160 (7)	0.0024 (6)	0.0061 (6)	0.0028 (6)
C10	0.0175 (8)	0.0242 (8)	0.0197 (8)	0.0028 (6)	0.0094 (6)	0.0010 (6)
C11	0.0129 (8)	0.0243 (8)	0.0231 (8)	0.0019 (6)	0.0076 (6)	0.0029 (7)
C12	0.0154 (8)	0.0197 (8)	0.0173 (8)	0.0000 (6)	0.0033 (6)	−0.0002 (6)
C13	0.0156 (8)	0.0176 (7)	0.0146 (7)	0.0011 (6)	0.0059 (6)	0.0013 (6)
C14	0.0136 (7)	0.0138 (7)	0.0152 (7)	0.0017 (5)	0.0048 (6)	0.0026 (6)
C15	0.0181 (8)	0.0198 (8)	0.0142 (7)	0.0003 (6)	0.0061 (6)	−0.0021 (6)
C16	0.0197 (8)	0.0295 (9)	0.0177 (8)	−0.0019 (7)	0.0016 (6)	−0.0054 (7)

### Geometric parameters (Å, °)

**Table:**

O1—C3	1.3679 (19)	C7—C8	1.351 (2)
O1—C15	1.4273 (19)	C7—H7	0.949 (10)
O2—C4	1.3615 (19)	C8—C9	1.436 (2)
O2—H2O	0.848 (10)	C8—H8	0.951 (9)
O3—C11	1.372 (2)	C9—C10	1.417 (2)
O3—H3O	0.849 (10)	C9—C14	1.414 (2)
O4—C12	1.363 (2)	C10—C11	1.357 (2)
O4—C16	1.432 (2)	C10—H10	0.959 (10)
C1—C6	1.414 (2)	C11—C12	1.420 (2)
C1—C2	1.421 (2)	C12—C13	1.371 (2)
C1—C14	1.451 (2)	C13—C14	1.422 (2)
C2—C3	1.374 (2)	C13—H13	0.946 (9)
C2—H2	0.947 (9)	C15—H151	0.954 (10)
C3—C4	1.420 (2)	C15—H152	0.948 (9)
C4—C5	1.367 (2)	C15—H153	0.959 (9)
C5—C6	1.414 (2)	C16—H161	0.955 (10)
C5—H5	0.954 (9)	C16—H162	0.952 (10)
C6—C7	1.431 (2)	C16—H163	0.959 (10)
			
C3—O1—C15	116.73 (12)	C10—C9—C8	120.47 (15)
C4—O2—H2O	108.9 (19)	C14—C9—C8	119.79 (14)
C11—O3—H3O	105 (2)	C11—C10—C9	120.43 (15)
C12—O4—C16	117.34 (13)	C11—C10—H10	119.4 (14)
C6—C1—C2	118.23 (14)	C9—C10—H10	120.1 (14)
C6—C1—C14	119.23 (14)	C10—C11—O3	120.48 (16)
C2—C1—C14	122.54 (14)	C10—C11—C12	120.56 (15)
C3—C2—C1	120.55 (14)	O3—C11—C12	118.95 (15)
C3—C2—H2	118.9 (13)	O4—C12—C13	127.02 (15)
C1—C2—H2	120.6 (13)	O4—C12—C11	112.88 (15)
O1—C3—C2	125.36 (14)	C13—C12—C11	120.09 (15)
O1—C3—C4	113.75 (14)	C12—C13—C14	120.56 (15)
C2—C3—C4	120.89 (14)	C12—C13—H13	119.0 (13)
O2—C4—C5	119.84 (15)	C14—C13—H13	120.4 (13)
O2—C4—C3	120.83 (14)	C9—C14—C13	118.58 (14)
C5—C4—C3	119.33 (15)	C9—C14—C1	119.15 (14)
C4—C5—C6	120.91 (15)	C13—C14—C1	122.27 (14)
C4—C5—H5	117.9 (13)	O1—C15—H151	103.7 (14)
C6—C5—H5	121.1 (13)	O1—C15—H152	108.2 (13)
C5—C6—C1	120.07 (14)	H151—C15—H152	114.5 (19)
C5—C6—C7	120.38 (14)	O1—C15—H153	112.9 (12)
C1—C6—C7	119.55 (14)	H151—C15—H153	109.3 (19)
C8—C7—C6	121.47 (15)	H152—C15—H153	108.3 (18)
C8—C7—H7	121.1 (13)	O4—C16—H161	106.3 (14)
C6—C7—H7	117.4 (13)	O4—C16—H162	111.6 (15)
C7—C8—C9	120.69 (15)	H161—C16—H162	112 (2)
C7—C8—H8	121.4 (13)	O4—C16—H163	106.8 (15)
C9—C8—H8	117.9 (13)	H161—C16—H163	112 (2)
C10—C9—C14	119.73 (15)	H162—C16—H163	108 (2)
			
C6—C1—C2—C3	0.0 (2)	C14—C9—C10—C11	−0.4 (2)
C14—C1—C2—C3	−179.27 (14)	C8—C9—C10—C11	178.42 (16)
C15—O1—C3—C2	5.0 (2)	C9—C10—C11—O3	179.88 (15)
C15—O1—C3—C4	−174.81 (13)	C9—C10—C11—C12	−1.0 (3)
C1—C2—C3—O1	179.19 (14)	C16—O4—C12—C13	0.9 (3)
C1—C2—C3—C4	−1.0 (2)	C16—O4—C12—C11	−179.78 (15)
O1—C3—C4—O2	−0.1 (2)	C10—C11—C12—O4	−178.39 (16)
C2—C3—C4—O2	−179.89 (14)	O3—C11—C12—O4	0.7 (2)
O1—C3—C4—C5	−179.60 (14)	C10—C11—C12—C13	1.0 (3)
C2—C3—C4—C5	0.6 (2)	O3—C11—C12—C13	−179.95 (15)
O2—C4—C5—C6	−178.64 (14)	O4—C12—C13—C14	179.82 (16)
C3—C4—C5—C6	0.9 (2)	C11—C12—C13—C14	0.6 (2)
C4—C5—C6—C1	−1.9 (2)	C10—C9—C14—C13	1.9 (2)
C4—C5—C6—C7	177.63 (15)	C8—C9—C14—C13	−176.96 (14)
C2—C1—C6—C5	1.4 (2)	C10—C9—C14—C1	−178.18 (14)
C14—C1—C6—C5	−179.26 (14)	C8—C9—C14—C1	3.0 (2)
C2—C1—C6—C7	−178.10 (14)	C12—C13—C14—C9	−1.9 (2)
C14—C1—C6—C7	1.2 (2)	C12—C13—C14—C1	178.09 (15)
C5—C6—C7—C8	−177.65 (15)	C6—C1—C14—C9	−3.6 (2)
C1—C6—C7—C8	1.9 (2)	C2—C1—C14—C9	175.69 (14)
C6—C7—C8—C9	−2.5 (2)	C6—C1—C14—C13	176.38 (14)
C7—C8—C9—C10	−178.78 (15)	C2—C1—C14—C13	−4.3 (2)
C7—C8—C9—C14	0.0 (2)		

### Hydrogen-bond geometry (Å, °)

**Table:**

*D*—H···*A*	*D*—H	H···*A*	*D*···*A*	*D*—H···*A*
O2—H2o···O1	0.85 (1)	2.20 (3)	2.670 (2)	115 (2)
O2—H2o···O3^i^	0.85 (1)	1.95 (1)	2.754 (2)	159 (3)
O3—H3o···O4	0.85 (1)	2.08 (3)	2.614 (2)	121 (3)

Symmetry codes: (i) *x*+1, *y*, *z*.
